# IsoFishR: An application for reproducible data reduction and analysis of strontium isotope ratios (^87^Sr/^86^Sr) obtained via laser-ablation MC-ICP-MS

**DOI:** 10.1371/journal.pone.0204519

**Published:** 2018-09-27

**Authors:** Malte Willmes, Katherine M. Ransom, Levi S. Lewis, Christian T. Denney, Justin J. G. Glessner, James A. Hobbs

**Affiliations:** 1 Department of Wildlife, Fish and Conservation Biology, UC Davis, Davis, California, United States of America; 2 Department of Land, Air, and Water Department, UC Davis, Davis, California, United States of America; 3 Interdisciplinary Center for Plasma Mass Spectrometry, UC Davis, Davis, California, United States of America; University of Otago, NEW ZEALAND

## Abstract

The *IsoFishR* application is a data reduction and analysis tool for laser-ablation strontium isotope data, following common best practices and providing reliable and reproducible results. Strontium isotope ratios (^87^Sr/^86^Sr) are a powerful geochemical tracer commonly applied in a wide range of scientific fields and laser-ablation inductively coupled mass spectrometry is considered the method of choice to obtain spatially resolved ^87^Sr/^86^Sr isotope ratios from a variety of sample materials. However, data reduction and analyses methods are variable between different research groups and research communities limiting reproducibility between studies. IsoFishR provides a platform to standardize these methods and can be used for both spot and time-resolved line transects. Furthermore, it provides advanced data analysis tools and filters for outlier removal, noise reduction, and visualization of time resolved data. The application can be downloaded from GitHub (https://github.com/MalteWillmes/IsoFishR) and the source code is available, encouraging future development and evolution of this software.

## Introduction

Strontium is a lithophile alkaline earth metal and substitutes easily for Ca^2+^ in the structures of minerals such as plagioclase feldspar, gypsum, calcite, dolomite, aragonite and apatite [1]. As a trace element, it is found in many igneous, metamorphic and sedimentary rock types, in the ocean, rivers and groundwater, in soils, plants, and animal soft and hard tissues. It has four naturally occurring isotopes, all of which are stable, namely ^84^Sr (~0.56%), ^86^Sr (~9.87%), ^87^Sr (~7.04%) and ^88^Sr (~82.53%) [2]. The isotopic abundance of ^87^Sr varies because of the production of radiogenic ^87^Sr by the decay of ^87^Rb by emission of a negative *β*-particle with a half-life of ~4.88 x 10^10^ years (Eq 1).

$$\frac{87}{37}Rb\to \frac{87}{38}Sr+\beta^{-}+\underline{v}+Q$$ (1)

***β***^−^beta particle

$\underline{v}$ antineutrino

Q decay energy

^87^Sr/^86^Sr composition of materials found in nature can vary based on the age and Rb/Sr composition of the underlying bedrock geology. Through weathering, this bedrock strontium is transferred into the environment: the soils, plants, and waterbodies of a region. Thus, the ^87^Sr/^86^Sr isotope ratios measured in minerals and tissues such as teeth, bones, otoliths, shells, feathers, and speleothems, reflect the available sources of strontium that were present during their formation. This also applies to geologically derived materials, such as pottery or flint, that were sourced locally. Consequently, it can be possible to trace the origin of materials and reconstruct a time resolved record of movement and transport across geologically different regions.

Due to the natural variation in strontium isotope ratios (^87^Sr/^86^Sr), they are a widely applied geochemical tracer across scientific fields including geology [3,4], ecology [5–7], paleoclimate sciences [8,9], archaeology [10,11], food sciences [12,13], and forensic sciences [14].

Laser-ablation (LA) mass spectrometry is considered the method of choice for many of these applications because it provides a high spatial sampling resolution (on the order of ~10s of μm) and a high sample throughput. The analytical methods to apply laser-ablation to a variety of natural materials were developed early on [15–17] and are constantly evolving and improving [18–24]. Recently, large amounts of effort have been placed on producing accurate and reliable data even from analytically problematic materials, such as bioapatite, that have direct interferences on the ^87^Sr/^86^Sr isotope ratio [25–27]. However, with a few exceptions such as the commercial program “Iolite” [28,29], software developed to process and analyze ^87^Sr/^86^Sr isotope ratios has been limited. Consequently, data reduction and data analyses methods are variable between laboratories, making direct data comparisons and aggregation challenging. Furthermore, evaluating the quality of strontium isotope data relies on proper reporting of data reduction procedures, interference corrections, and analyses parameters [19,21,26]. Finally, most conventional software is geared towards non-time resolved spot analysis, where each analysis is treated as a single homogenous sample. However, one of the great advantages of laser ablation analysis is the time-resolved continuous track method, where the laser traverses across a heterogeneous sample to identify changes in the ^87^Sr/^86^Sr isotope ratio at a high spatial resolution. In the case of continuous track analysis, a single averaged ^87^Sr/^86^Sr value is not appropriate, rather the full time-resolved data profile is of interest. This is also important for applications using laser-ablation for depth profiling, where the laser is used to drill into the sample to obtain a time-resolved record from the outside of the sample without prior cutting [18,30].

To address the need for consistent data reduction and reporting as well as useful analysis of continuous track laser-ablation mass spectrometry ^87^Sr/^86^Sr data, we developed the IsoFishR application. The aim of this application is to provide rapid, reliable, and reproducible data reduction and analysis of laser-ablation strontium isotope ratios, that can be applied across different scientific disciplines and sample materials. In contrast to a software such as Iolite, that is aimed at analyzing a whole range of different element ratios and isotopes, our tool is specific to strontium isotopes (^87^Sr/^86^Sr) which allows a focused and targeted solution for high sample throughput. Additionally, our software is open source, so it can be adapted by its users to fit their data reduction and data analyses preferences.

## Program architecture

### Overview

The IsoFishR application is written in the statistical programming language R, which is an open source language that is supported across all major operating systems [31]. R was chosen because it will ensure the usability and longevity of the application due to the large amounts of data manipulation and statistical packages already available, including packages directly aimed at isotope and elemental analyses [32–35]. The backbone of the IsoFishR application is the R package shiny [36], which allows for the creation of an interactive graphical user interface (GUI) connected to a running R session. This allows the user to perform complex operations without directly having to interact with the underlying R commands. IsoFishR utilizes several other established R packages (Table 1), which are available on the Comprehensive R Archive Network; CRAN (http://cran.rproject.org) and are installed and loaded automatically when running IsoFishR. The source code is maintained using Git version control and hosted online (https://github.com/MalteWillmes/IsoFishR), which allows for issue tracking, and development and dissemination of new features and updates. In GitHub, users can create their own branch of the application and modify it to fit their study protocols while staying connected to any improvements made to the base version of the application. This paper describes the master branch, stable release version 1.0. Changes to the application will be reflected in the version number and listed in the readme document on GitHub.

**Table 1:** List of the R packages currently utilized by IsoFishR. More information for each package is available on their respective CRAN sites or on GitHub.

R package name	Use	Reference
‘shiny'	A web application framework for R	[36]
‘shinydashboard',	Creating dashboards in shiny	[37]
‘shinyWidgets'	Custom inputs widgets	[38]
‘tidyverse'	Collection of compatible R packages for advanced data cleaning, manipulation, and plotting	[39]
‘broom’	Convert statistical objects into tidy data frames	[40]
‘changepoint'	Changepoint analysis	[41]
‘colourpicker'	Color picking for shiny	[42]
‘mgcv'	Generalized Additive Models	[43]
‘DT’	Data objects and data table in R	[44]
‘zoo'	Advanced moving average functions	[45]

### Layout

The IsoFishR application is divided into a navigation menu (left sidebar) and a main window that displays the different pages. Five pages can be accessed through the navigation window; About, Projects, Data Reduction, Data Analysis, and Data Reporting.

**Fig 1:**
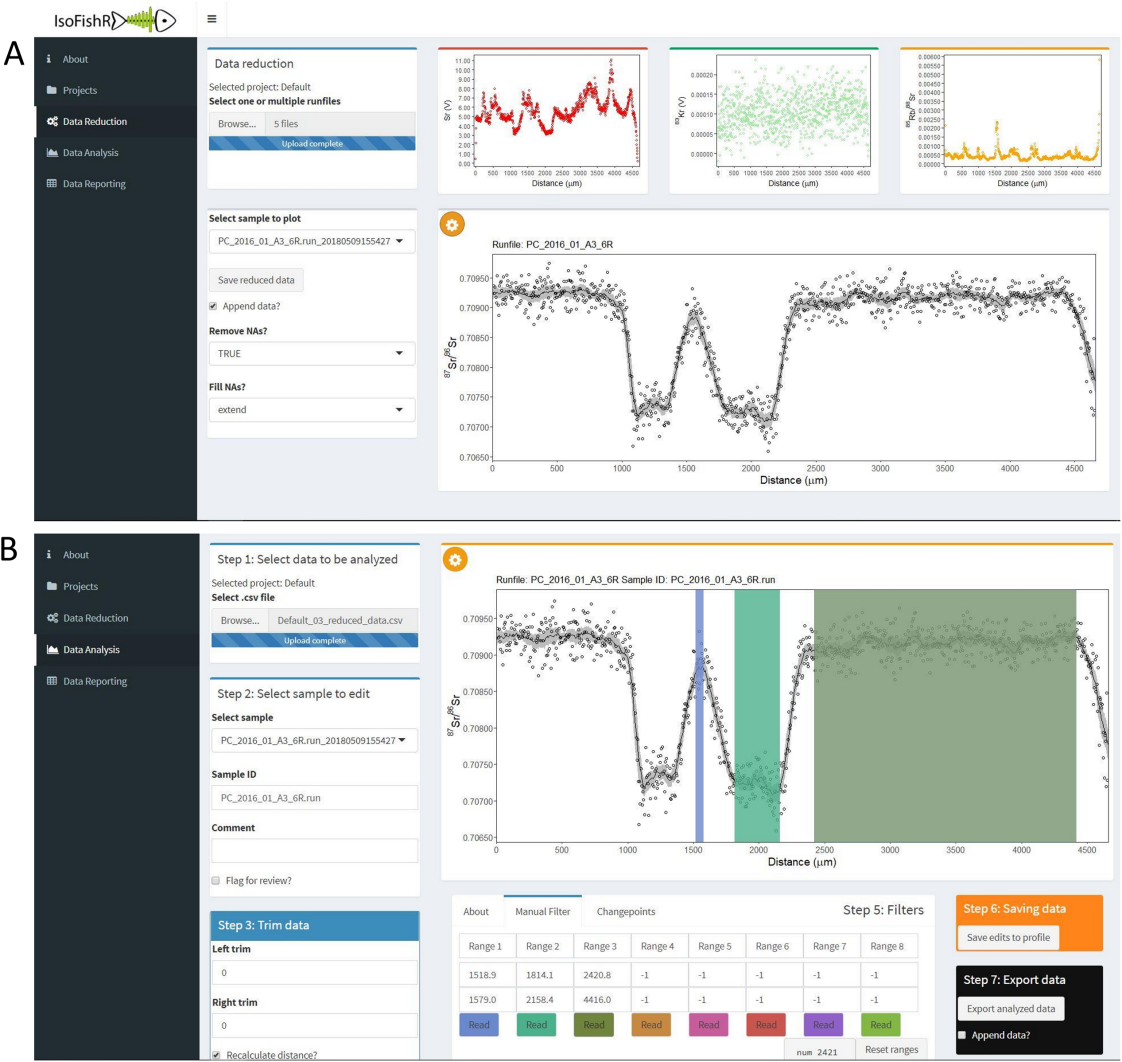
Screen shot of the IsoFishR application. (A) shows the “Data Reduction” page selected and (B) the “Data Analysis” page selected, and three manual filters applied. The ^87^Sr/^86^Sr data currently loaded is a line transect across a Chinook salmon (*Oncorhynchus tshawytscha*) otolith from the example data provided.

The “About” page consists of a short introduction section and describes the functionality of the application, including links to the tutorial, GitHub issue tracker, as well as funding sources, and license information. The “Projects” page allows users to create and manage projects. Projects are used in the application to group similar types of analyses together (for example a set of samples). They define the import settings from the mass spectrometer and set the default data reduction parameters to apply to each sample. Creating a new project creates a set of folders in the app root directory where all settings, data, and plots are stored. The “Data Reduction” page (Fig 1A) is used to process the data files from the mass spectrometer and to provide a quick overview of the data quality. After the data has been reduced it can be edited and manipulated using the “Data Analysis” page (Fig 1B). Finally, the “Data Reporting” page provides a quick overview of the current analysis.

### Features

**Fig 2:**
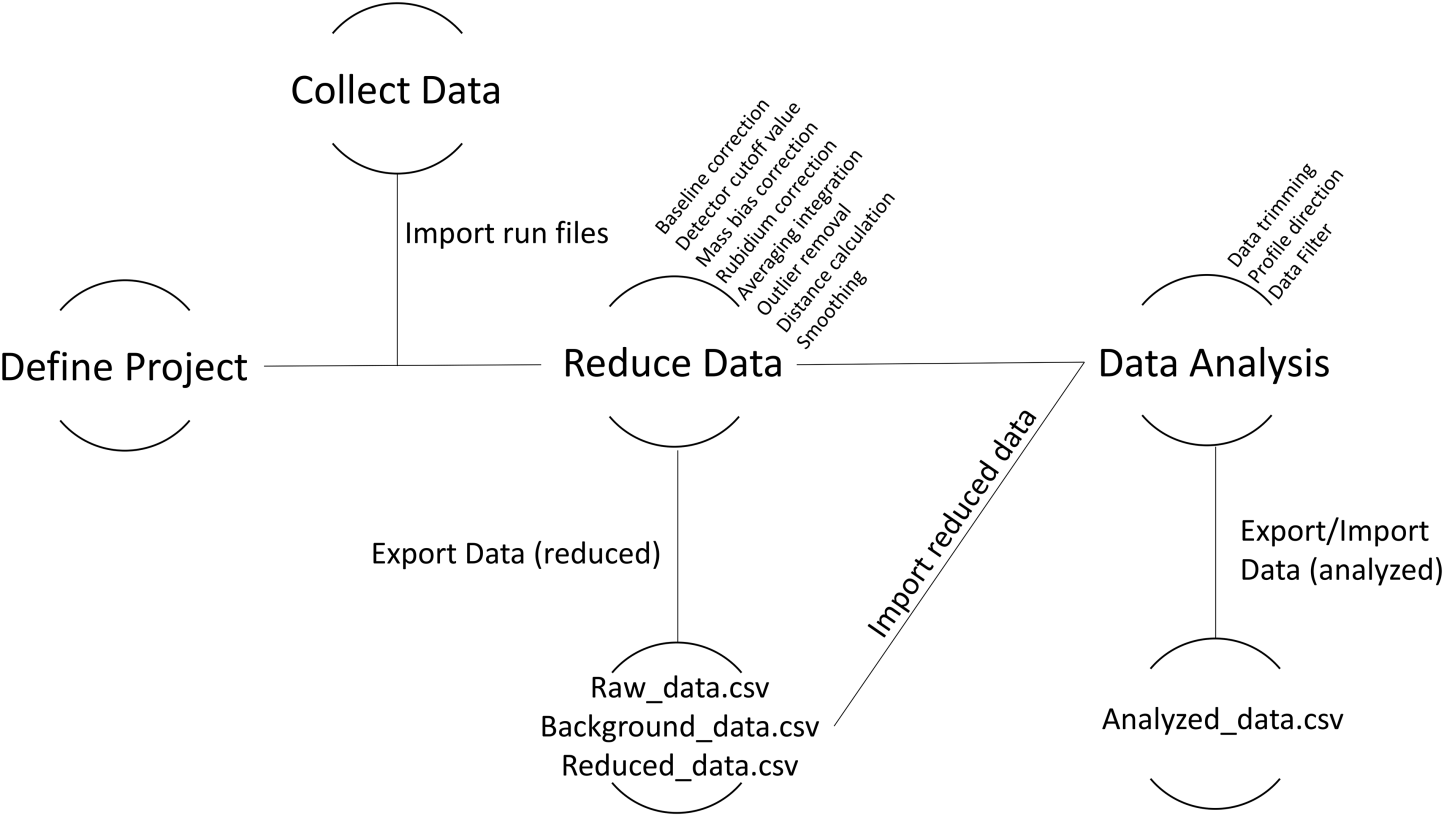
Standard workflow overview. Data reduction and analysis parameters are first defined in the “Define Project” stage and then consequently applied. Data can be exported both after the data reduction and after the data analysis phase.

The standard workflow is shown in Fig 2 and the following section explains the available features of the IsoFishR application (version 1.0) in detail. Data are imported in the “Data Reduction” page and multiple files from a single folder can be selected for import. There is no limit to the individual data files that make up a project but loading times increase significantly past ~500 individual sample files. Once data has been reduced it can be imported in the “Data analysis” page and can be reimported multiple times as long as the original columns are not changed (e.g. outside of R). This allows the user to work on data analysis iteratively and save the changes. The project and datafiles include all the relevant analysis information so that a project can be transferred between different users or could be included as supplementary material in publications which would allow any user to recalculate and evaluate the presented data.

### Data import

Data import format, such as the number and order of columns in the mass spectrometer export file, varies between different mass spectrometers, labs, and projects. For data reduction in IsoFishR several specific measurements are required (Table 2). To facilitate the import of different data files into the IsoFishR application the project page allows the user to change and select the order of columns and the format of the original data file. Once a new data import format has been developed it is saved together with the project file and is available for any future analyses.

**Table 2:** Example format of raw data from the Nu CAMECA mass spectrometer export file at UC Davis. Order of columns, length of header, and separator type can be changed within the project settings. “Cycle Number” (column 7) is not required. The import format can be adjusted to accommodate other instrument method files in the project page.

1	2	3	4	5	6	7	8
Raw88 (V)	Raw87 (V)	Raw86 (V)	Raw85 (V)	Raw84 (V)	Raw83 (V)	Cycle Number	Cycle Seconds

### Data reduction

Depending on the analyzed material, there are a number of potential isobaric interferences on the ^87^Sr/^86^Sr ratio including double charged rare earth elements (REEs), Kr, Rb, Ca dimers, Ca argides, and polyatomic interferences, see Table 3 [19,21,46]. Data reduction procedures in IsoFishR follow established protocols for Kr and Rb corrections using known isotope ratios [47]. No corrections are currently applied for interferences from rare earth elements, Ca dimers, Ca argides, or polyatomic interferences. These interferences are complex and occur only in some sample materials (e.g., biogenetic apatite) and consequently are not included in the default data reduction schema. For these applications the interferences could be monitored (e.g., ^89^Y for REEs), and corrected as needed by either peak stripping (Ca dimers, Ca argides) or controlled analytical conditions and instrument modifications (polyatomic interferences). The Ca dimer and Ca argide peak stripping could be a valuable extension of the current data reduction procedure, because while it does not significantly affect the ^87^Sr/^86^Sr isotope ratio in most applications, it does lead to a shift in the ^84^Sr/^86^Sr isotope ratio, which can be used as a monitor for data quality [19,21,46].

**Table 3:** Common interferences on ^87^Sr/^86^Sr isotope ratios, table adapted from [21,48].

Mass		82	83	84	85	86	87	88	89
Strontium				^84^Sr		^86^Sr	^87^Sr	^88^Sr	
Krypton	Baseline subtraction	^82^Kr	^83^Kr	^84^Kr		^86^Kr			
Rubidium	Peak stripping				^85^Rb		^87^Rb		
REEs	-	Er^2+^ Dy^2+^	^166^Er^2+^	^168^Yb^2+ 168^Er^2^	^170^Yb^2+ 170^Er^2+^	^172^Yb^2+^	^174^Yb^2+^	^176^ Lu^2+^ ^176^Yb^2+^ ^176^Hf^2+^	^89^Y
Ca dimers	-	^40^Ca^42^Ca	^40^Ca^43^Ca	^40^Ca^44^Ca	^42^Ca^43^Ca	^40^Ca^46^Ca ^42^Ca^44^Ca	^43^Ca^44^Ca	^40^Ca^48^Ca	
Ca argides	-	^40^Ar^42^Ca	^40^Ar^43^Ca	^40^Ar^44^Ca		^40^Ar^46^Ca	^43^Ca^44^Ca	^40^Ar^48^Ca	
Polyatomic interferences	-						^40^Ca^31^P^16^O ^40^Ar^31^P^16^O

1. *Averaging integration*: The first step is to use the “integration time” value to average the raw data to a one data point per second resolution. For example, an integration time set to five indicates that the instrument collected five raw data points per second (0.2s per raw data point) and the application will average these to a single value.
2. *Baseline correction*: Correcting for the krypton interference originating from the argon supply (^86^Kr) is achieved by monitoring the background level before the analysis and subtracting the background from the data. In the default configuration, the data background is defined as any value below a user defined mass 88-voltage (V) cutoff, default is set to 0.05 V during a set blank time at the beginning of the measurement, default is 30 seconds.
3. *Detector cutoff value*: A maximum 88 V cutoff is applied to the entire data to exclude any values that went over voltage, default 9.8 V. This should be adjusted to the detectors used on the instrument.
4. *Mass bias correction*: The mass bias induced from the laser and the instrument mass discrimination is calculated using an exponential correction to the stable ^86^Sr/^88^Sr ratio of 0.1194 (Eqs 2 and 3).
  (Sr87Sr86)corrected=(Sr87Sr86)measured×(m87m86)f (2)
  **f** Sr mass bias
  *m*_87_ 86.9088775
  *m*_86_ 85.9092607
  f=ln[(Sr86Sr88)true(Sr86Sr88)measured]ln(m86m88) (3)
  (Sr86Sr88)true 0.1194
  *m*_88_ 87.9056123
5. *Rubidium correction*: The direct interference of ^87^Rb on the ^87^Sr/^86^Sr isotope ratio is corrected by monitoring ^85^Rb and subtracting the appropriate amount from the signal at mass 87 assuming the natural ^85^Rb/^87^Rb ratio of 2.59712. The ^85^Rb/^87^Rb ratio is corrected for mass bias, using the mass discrimination factor calculated from strontium (Eqs 4 and 5). Next, the strontium mass bias is applied to the Rb corrected ^87^Sr/^86^Sr isotope ratio from Eq 5 using Eqs 2 and 3.
  Rb87measured=(Rb87Rb85)true×Rb85measured(m87m85)f (4)
  *m*_85_ 84.9117897
  *m*_87_ 86.9091805
  Sr87measured=87Intensity-Rb87measured (5)
6. *Outlier detection and removal*: Outliers in the data are the result of potentially many different sources such as surface features, cracks, or residues from resins, dust, or other materials. They are removed based on the median and a multiplicate of the standard deviation, default is 2, calculated over a user defined moving median window.
7. *Distance calculation*: The run speed value is used to calculate the length of the analysis based on the time of the analysis. This results in a distance resolved ^87^Sr/^86^Sr isotope profile as shown in the main plot window on the “Data Reduction” page.
8. *Smoothing*: Laser-ablation strontium isotope data is typically accompanied by more signal noise than traditional solution-based work both from instrumental conditions (laser pulses, gas uptake), as well as actual changes within the sample’s physical properties and composition (e.g., zones within an otolith). Statistical smoothing of reduced data has the aim to minimize noise while keeping the important changes in the profile. This is achieved in IsoFishR using a moving average or applying a thin plate spline fit. Both functions allow the user to vary the degree of smoothing appropriate for different instruments and sample materials.
9. At this stage data can be saved (either appending to an existing project or overwriting any present files) and moved outside of the IsoFishR application for further analysis or imported into the “Data analysis” page.

### Data analysis

First the reduced data needs to be reimported (e.g., Project_name_03_reduced_data.csv). Then a variety of data analysis options are available, and they should be carried out in the sequence as denoted on the “Data Analysis” page.

#### Data selection

Trimming of data is a common task as the laser ablation analysis is time dependent and sometimes starts and/or ends outside of the bounds of the sample material. Additionally, the laser and mass spectrometer are often given time to stabilize before collecting important data. Trimming at the beginning of the profile will recalculate the distance based on the new zero position (if enabled), while trimming at the end of the profile will remove the entered distance in μm from the end of the profile. Since trimming removes data from the analysis file it is important to perform the trimming step before doing any further qualitative or quantitative data analysis.

#### Profile orientation

Next the profile can also be reversed (inverting the x-direction of the analysis). This can be useful if some of the profiles were analyzed in different directions, but the same orientation is required for further analysis.

#### Data filtering

The IsoFishR application has three options for calculating summary statistics of the distance resolved ^87^Sr/^86^Sr isotope profile: (i) no distinct defined regions (homogenous sample case), (ii) user defined manual regions, and (iii) statistically defined regions.

(i) Homogenous sample, for example a reference material, a simple average across the trimmed data is most appropriate and this is automatically calculated for any analyzed sample.
(ii) Heterogeneous types of samples require different approaches. For example, in a case where the sample material has a distinctive physically observable pattern (such as the rings in an otolith, or layers in a speleothem), performing summary statistics within distinct regions along the profile may be more appropriate. Within IsoFishR, regions can be manually defined using the known distances from the start of the profile.
(iii) In other cases, it may be more appropriate to determine distinct ranges within a sample based on some statistical test. In IsoFishR this can be achieved by implementing changepoint analysis [41]. The changepoint analysis is a statistical approach in which the variability in the ^87^Sr/^86^Sr isotope ratio along the profile is used to define significant regions. IsoFishR calculates summary statistics for sample points that fall within each user defined or changepoint defined region. There are many other pattern detection methods available in R that can easily be implemented in IsoFishR, if required.

#### Saving analyzed data

Changes to each profile are saved using the “save edits to profile button” and at the end of the data analysis the complete data can be exported using the “Export analyzed data” option. Previously analyzed data can be reimported and edited.

### Data reporting

The analyzed data from IsoFishR is saved in “long” format, where each row is one observation of the sample. This allows for easy data transfer for additional analyses either in R or python. More extensive data analyses and reporting should be done outside of IsoFishR (see next section on data exporting). However, for a quick overview the “Data Reporting” tab can be used. It includes a list of data tables and a grouping tab for creating a summary data table.

### Data export

Data is exported as comma delimited (.csv) files which can be imported into a variety of software programs (including R) for further data analysis. For each step and each analyzed profile, the application saves to a summary file and creates plot files in PDF format. Data exports are organized in tiers, starting with raw data at T1, background data T2, Reduced data T3 from the “Data Reduction” page, and analyzed data T4 from the “Data Analysis” page. A metadata file is included in the application folder which contains the detailed descriptions for each column of data.

## Example data

### Line scan/transect analysis

**Fig 3:**
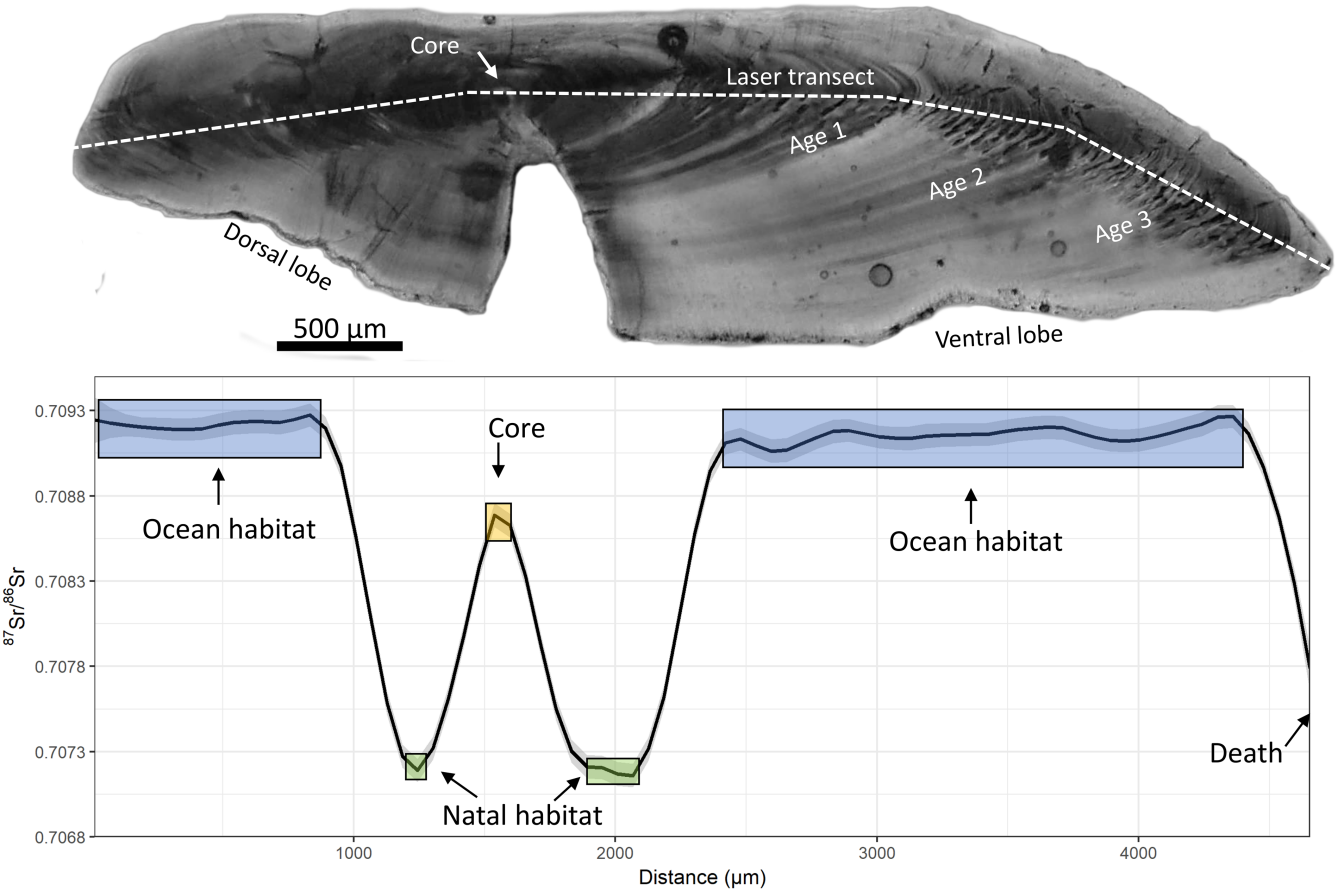
An image of a Chinook salmon (*Oncorhynchus tshawytscha*) otolith and the corresponding ^87^Sr/^86^Sr isotope profile. The otolith was aged, and microstructures were visually identified to inform the selection of discrete ranges within the strontium isotope profile that relate to different life stages of the salmon.

Here we include a case study to illustrate the work flow for a typical application in IsoFishR. The case study uses ^87^Sr/^86^Sr isotope analysis of otoliths (“earbones”) to identify the life history of a Chinook salmon (*Oncorhynchus tshawytscha*) from the Central Valley, California. Otoliths consist of calcium carbonate (aragonite) and accrete continuously throughout the life of a fish, providing a life-long archive of physiological and environmental conditions. The accretions typically form banded microstructures that are identifiable under a microscope and are commonly used for aging the fish. This physical information can be used to filter the strontium isotope data obtained from laser ablation analyses (Fig 3).

The five sagittal otoliths provided in this case study were collected in 2016 during carcass surveys on Putah Creek, a small river between UC Davis and the city of Winters, CA. Salmon were previously extirpated from this river and consequently identifying their origin is of great interest. The example data analysis encompasses the following steps:

Step 1) Create a new project called “Putah Creek Salmon” and adjust the default data reduction settings. The otoliths were analyzed using a run speed of 5 μm/s and a moving average of 20 points. The otoliths were analyzed from the dorsal to the ventral lobe capturing the full life history in duplicate. Update the project settings and add a comment (“Initial Putah Creek data reduction”).
Step 2) From the “Data reduction” page it is now possible to import the five runfiles, located in the “Example runfiles” folder within the app directory. Data reduction should only take a few seconds for this example data but can take much longer for a large number of raw data files.
Step 3) The sample selector tool enables the user to view each of the sample profiles individually. For an initial idea of the data quality, measurement parameters such as Sr (V), ^83^Kr, and ^85^Rb/^88^Sr plots are displayed and can be checked. The button next to the main ^87^Sr/^86^Sr graph enables various graphing options. Next, clicking on the “save reduced data” button will create three .csv files (Project_name_01_raw_data; Project_name_02_background_data; Project_name_03_reduced_data), as well as four plot types for each analyzed otolith (^83^Kr, ^85^Rb^88^Sr, ^87^Sr^86^Sr_reduced, and totalSr). At this stage further processing could be performed outside of IsoFishR or we can continue with data reduction and analysis within the app.
Step 4) Change to the “Data Analysis” page and import the Project_name_03_reduced_data.csv file. After the data is loaded individual samples can be selected with the sample selector. These example otoliths require data trimming to remove erroneous data points due to laser ablation analysis of the sample holder at the beginning and the end of the transect. A trim distance of 10 μm from both the left and right removes the erroneous data points for all samples except “PC_2016_01_B4_11R” which requires a 40 μm left trim. Trimming is either subjectively based on the quality of the data or can be estimated from measuring the laser scar under the microscope after the analyses.
Step 5) Adult salmon otoliths contain a core, a natal region, and an adult region. The core often shows a maternal influence (from the parent maturing in the ocean), the natal region should reflect the stream the fish spawned and reared in, and the adult region should reflect the ocean habitat. This ocean habitat is particular useful because the global marine ^87^Sr/^86^Sr ratio is well known at 0.70918 [49,50]. Consequently, this can be used as an internal reference in each of the samples. The core is visible under the microscope and was measured for each fish. The natal region is often assumed to be at roughly ~150–200 μm from the core but can vary between individuals depending on their growth. The adult region encompasses the rest of the otolith. Each of these regions can be marked using the manual region selection (Fig 3) by typing in the numbers or selecting the ranges on the graph (Table 4). The adult region can be defined by either the remaining section of the otolith after the natal portion, or alternatively, only the ocean habitat that displays a stable ^87^Sr/^86^Sr ratio around 0.70918. The final step is to save edits to each graph and then export the analyzed data.

**Table 4:** Measured microstructure features on otolith images for the core and natal regions of the five Chinook salmon (*Oncorhynchus tshawytscha*) example otoliths. The adult regions for these examples are based on the section of otolith for which the strontium isotope profile remains stable at around 0.70918.

Sample ID	Left trim	Right trim	Core (μm)	Natal (μm)	Adult (μm)
PC_2016_01_A2_4R	10	10	1980–2030	2164–2684	2870–5654
PC_2016_01_A3_6R	10	10	1520–1570	1810–2145	2355–4444
PC_2016_01_B4_11R	40	10	1564–1614	1790–2204	2424–4590
PC_2016_01_C3_15R	10	10	1525–1585	2070–2185	2425–4490
PC_2016_01_C4_16R	10	10	1977–2027	2262–2707	2932–5646

### Spot data analysis

**Fig 4:**
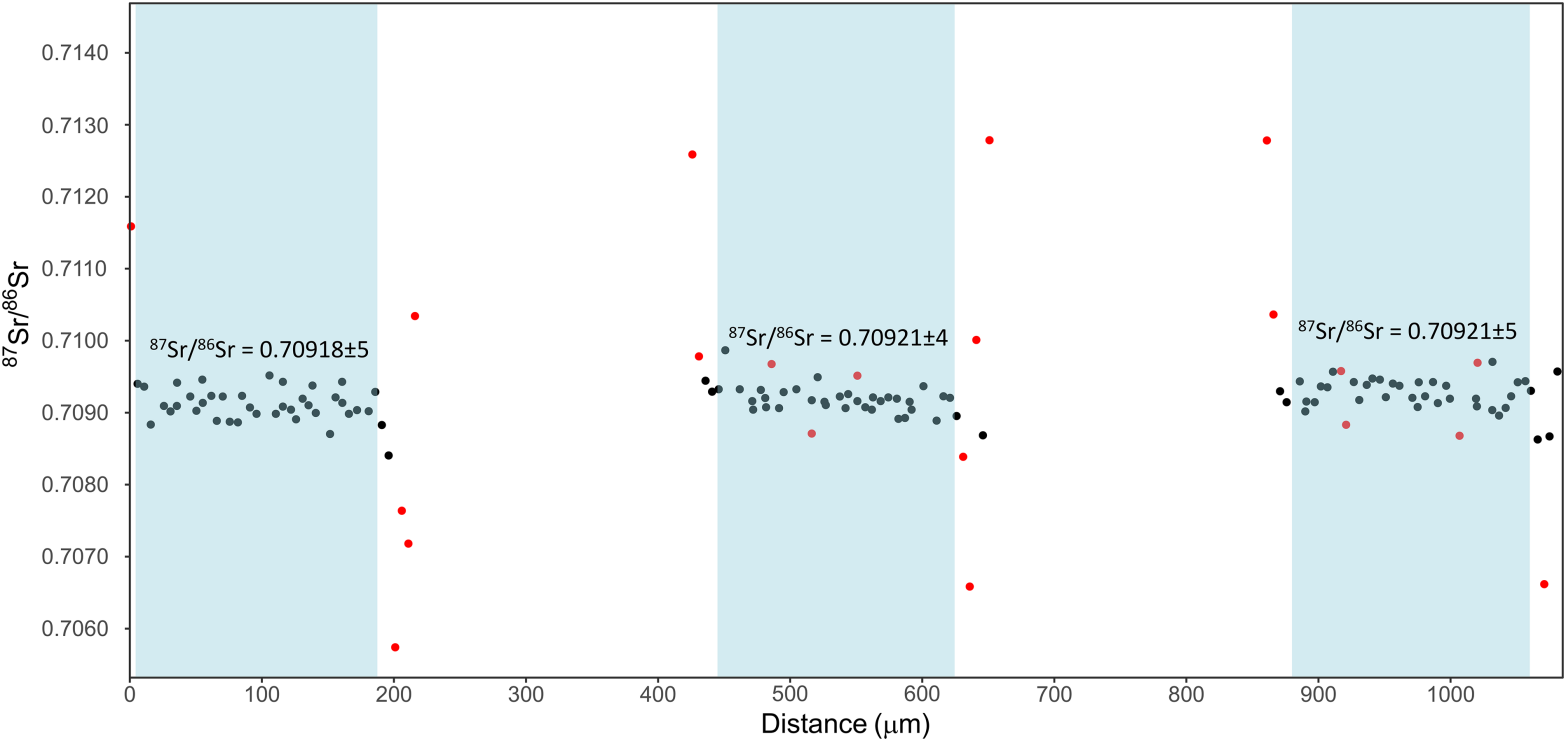
^87^Sr/^86^Sr results from 3 discrete spot analyses on the White Seabass (*Atractoscion nobilis*) otolith. Average values for each of the spots are shown and are in good agreement with the average ^87^Sr/^86^Sr ratio of modern ocean water (0.70918) [49,50]. The red data points indicate outlier values (2σ).

Spot data collection is also frequently used in laser ablation analysis. Here we include an example from one of our commonly used internal reference materials, a White seabass (*Atractoscion nobilis*) otolith. The white sea bass spends most of its life in the ocean and therefore any spot on the otolith from this fish should reflect the modern average global ocean ^87^Sr/^86^Sr ratio of 0.70918. For the example spot analysis create a new project named “reference_materials”, change the analysis type in the project settings to spot, and then click update project settings. Load the runfile in the “data reduction” tab. Here it is visible that multiple spot analyses were saved within a single run file and that the data reduction automatically picked out the correct areas to average over (Fig 4). Edits can then be exported and saved. The reduced data can be loaded in the “data analysis” tab for further processing, if needed, in order to trim and manually select spot regions.

## Conclusions and future directions

IsoFishR aims to provide a fast, reproducible, and transparent data reduction application for laser-ablation strontium isotope analysis. Since the application was programmed in R, it combines a robust framework with the ability for users to customize and adapt the program. Furthermore, many additional R packages for data smoothing and data filter processing are available and can be utilized for more advanced data exploration and analysis. We expect that the IsoFishR application will continue to evolve and we encourage contributions from other researchers and developers.

## Access to IsoFishR

IsoFishR is available under the MIT license from GitHub (https://github.com/MalteWillmes/IsoFishR) or by contacting Malte.willmes@googlemail.com. New versions of the application will be released on GitHub. Installation requires an up to date R installation. Please report any bugs and problems on GitHub.
